# Supplementary material for: Inherited C-terminal TREX1 variants disrupt homology-directed repair to cause senescence and DNA damage phenotypes in Drosophila, mice, and humans
Source: Nat Commun. 2024 Jun 1;15:4696. doi: 10.1038/s41467-024-49066-7 (PMC11144269; doi:10.1038/s41467-024-49066-7)
Supplement: Supplementary file 1 — Supplementary Information [file 41467_2024_49066_MOESM1_ESM.pdf]

SUPPLEMENTARY INFORMATION

Title: Inherited C-terminal TREX1 variants disrupt homology-directed repair to cause senescence and DNA damage phenotypes in *Drosophila*, mice, and humans

Authors: Samuel D. Chauvin[1,2,+], Shoichiro Ando[3,+], Joe A. Holley[1,2,+], Atsushi Sugie[4], Fang R. Zhao[5], Subhajit Poddar[1,2], Rei Kato[3], Cathrine A. Miner[1,2], Yohei Nitta[4], Siddharth R. Krishnamurthy[6,7], Rie Saito[8], Yue Ning[1,2], Yuya Hatano[3], Sho Kitahara[3], Shin Koide[3], W. Alexander Stinson[5], Jiayuan Fu[1,2], Nehalee Surve[1,2], Lindsay Kumble[1,2], Wei Qian[5], Oleksiy Polishchuk[1,2], Prabhakar S. Andhey[9], Cindy Chiang[10,25], Guanqun Liu[10,25], Ludovic Colombeau[11], Raphaël Rodriguez[11], Nicolas Manel[12], Akiyoshi Kakita[8], Maxim Artyomov[9], David C. Schultz[13], P. Toby Coates[14,15], Elisha D.O. Roberson[5], Yasmine Belkaid[6,7,16], Roger A. Greenberg[17], Sara Cherry[18,19], Michaela U. Gack[10,25], Tristan Hardy[20,21], Osamu Onodera[3,22], Taisuke Kato[22,*], Jonathan J. Miner[1,2,5,18,23,24,*]

[+,*]These authors contributed equally to this work.

List of Supplementary Materials

Supplementary Fig. 1. Disease-associated TREX1 mutants are stably expressed, but only RVCL mutants are mislocalized.

Supplementary Fig. 2. DDR signaling is up-regulated by TREX1 mutants associated with RVCL, but not by other TREX1 mutants associated with other diseases.

Supplementary Fig. 3. RVCL mutant TREX1 causes DNA damage via its exonuclease activity and nuclear localization.

Supplementary Fig. 4. Heterozygous RVCL mice express both full-length and C-terminally truncated TREX1.

Supplementary Fig. 5. Heterogeneity of TREX1 expression and sensitivity of RVCL cells to PARP1 inhibition or deletion.

Supplementary Fig. 6. Aberrant nuclear localization of TREX1 in primary bone marrow-derived macrophages from RVCL mice.

Supplementary Fig. 7. Sensitivity of RVCL cells and a patient with RVCL to aclarubicin.

Supplementary Figure 8. Interferon-stimulated gene expression in peripheral blood cells from patients with RVCL.

Supplementary Figure 9. Changes in *TREX1* and ISG expression in human tissues with age.

Supplementary Figure 10. Up-regulation of TREX1 with DNA-damaging agents.

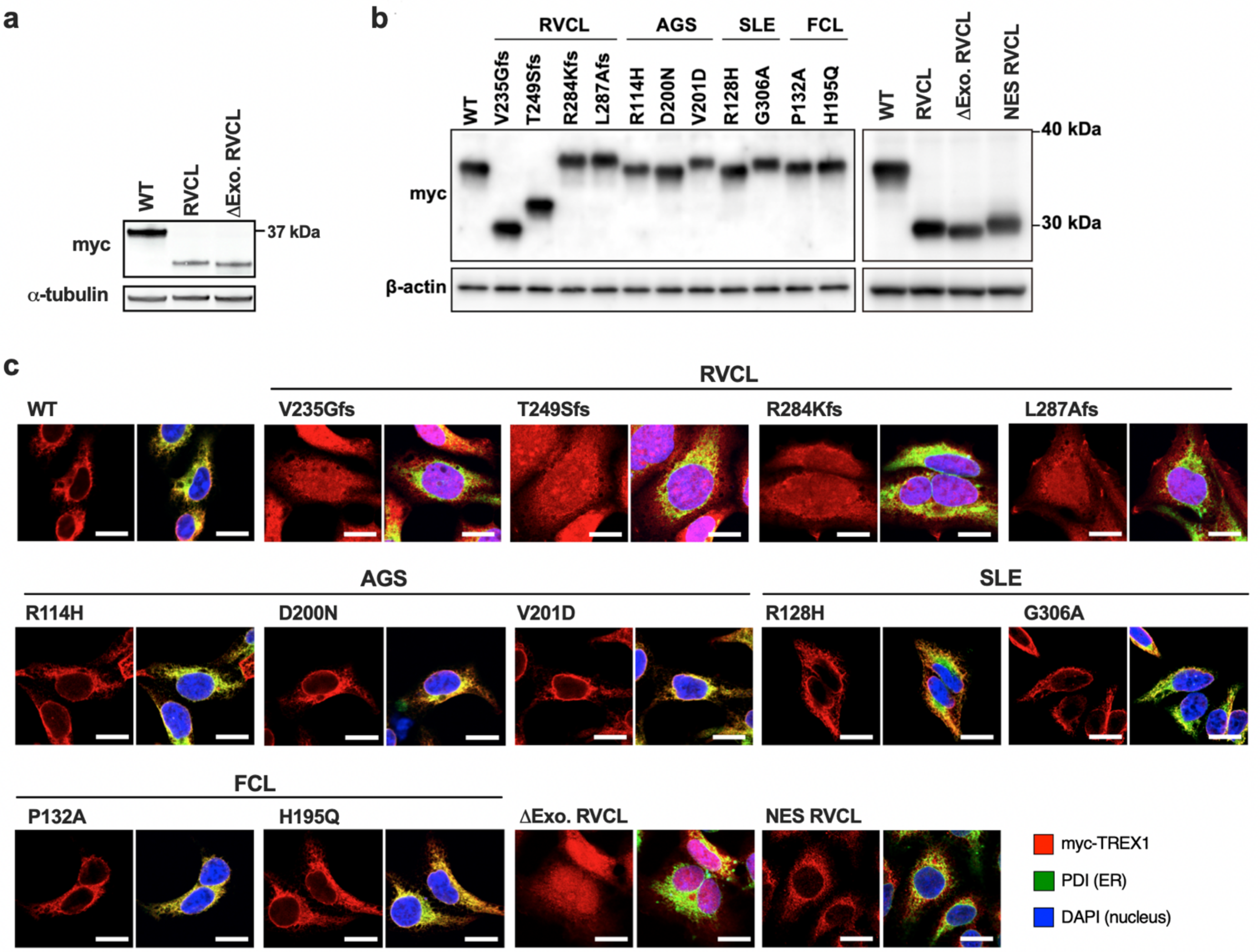

Supplementary Figure 1

Supplementary Figure 1. Disease-associated TREX1 mutants are stably expressed, but only RVCL mutants are mislocalized. (**a**) A representative Western blot showing expression of myc-tagged TREX1 in the eye of transgenic *Drosophila* with immunoblotting of for myc and α-Tubulin. (**b**) A representative Western blot of doxycycline-treated Flp-in 293T cells with inducible expression of myc-tagged TREX1 mutants associated with RVCL, AGS, SLE, or FCL or myc-tagged ΔExo RVCL (TREX1 R62A/V235fs) or NES RVCL (TREX1 V235Gfs with a nuclear export signal) mutant TREX1 with immunoblotting for myc and β-actin. (**c**) Representative fluorescence microscopy images of Flp-in 293T cells expressing myc-tagged disease-associated TREX1 variants with immunostaining for myc (TREX1), protein disulfide isomerase (PDI; ER), and DAPI (nucleus). Scale bar = 15 µm. Data are representative of 3 independent experiments. Source data are provided as a Source Data file.

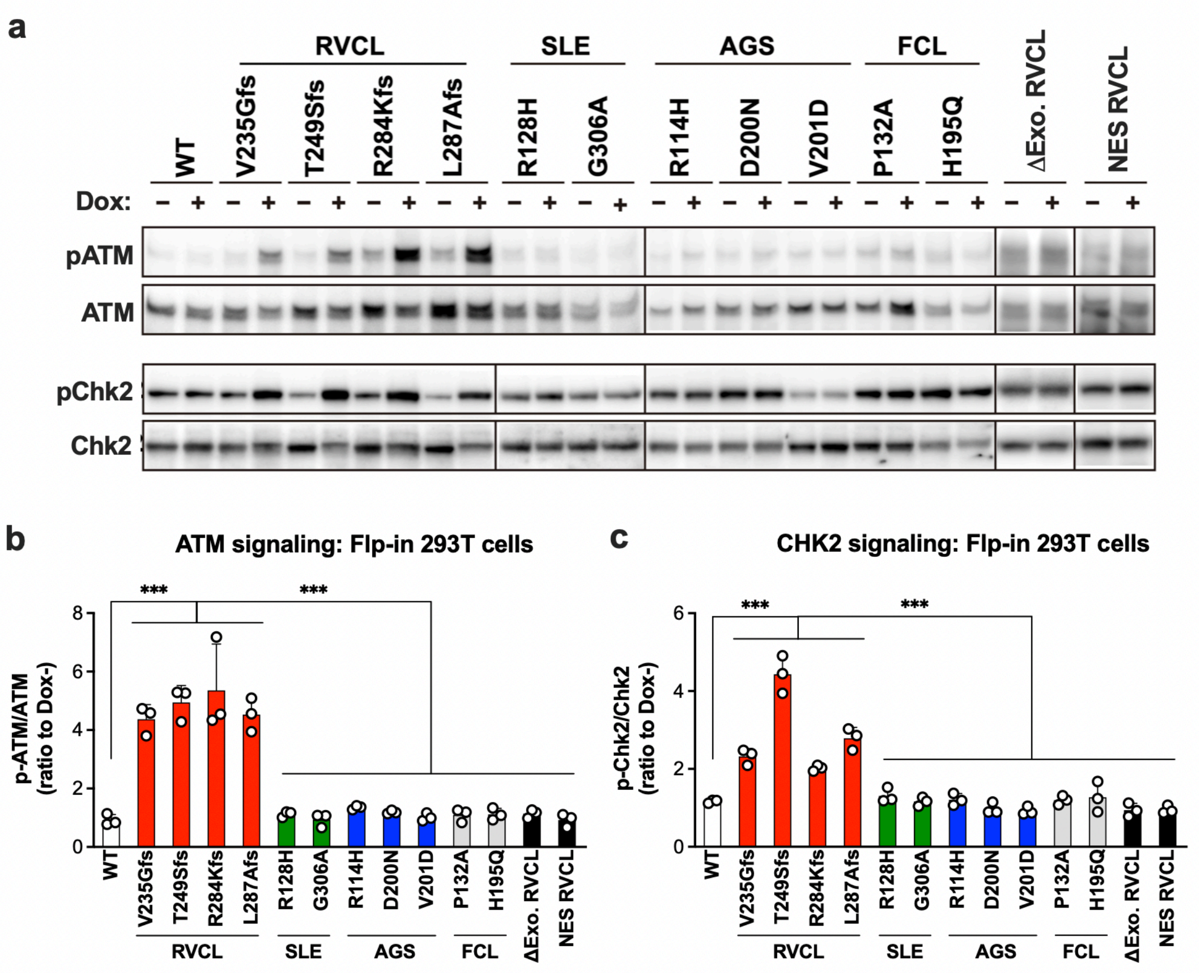

Supplementary Figure 2

Supplementary Figure 2. DDR signaling is up-regulated by TREX1 mutants associated with RVCL, but not by other TREX1 mutants associated with other diseases. (**a**) Representative Western blot of Flp-in293 cells before and after doxycycline-induced expression of WT TREX1, the indicated TREX1 mutants associated with RVCL, SLE, AGS, or FCL, or RVCL TREX1 (V235Gfs) with a nuclear export signal (NES RVCL) or lacking enzymatic activity (ΔExo RVCL), followed by immunoblotting for p-ATM, ATM, p-Chk2, and Chk2. Vertical lines in the image indicate that the images are from different blots. (**b–c**) Quantitation of the ratio of p-ATM to ATM (b) and p-Chk2 to Chk2 (c) from (a). Data in (a) are representative of 3 independent experiments. Data in (b–c) represent the mean ± the SD of n = 3 independent biological repeats. Data were analyzed by ANOVA with Bonferroni post hoc comparison. Source data are provided as a Source Data file.
*** $P < 0.001$.

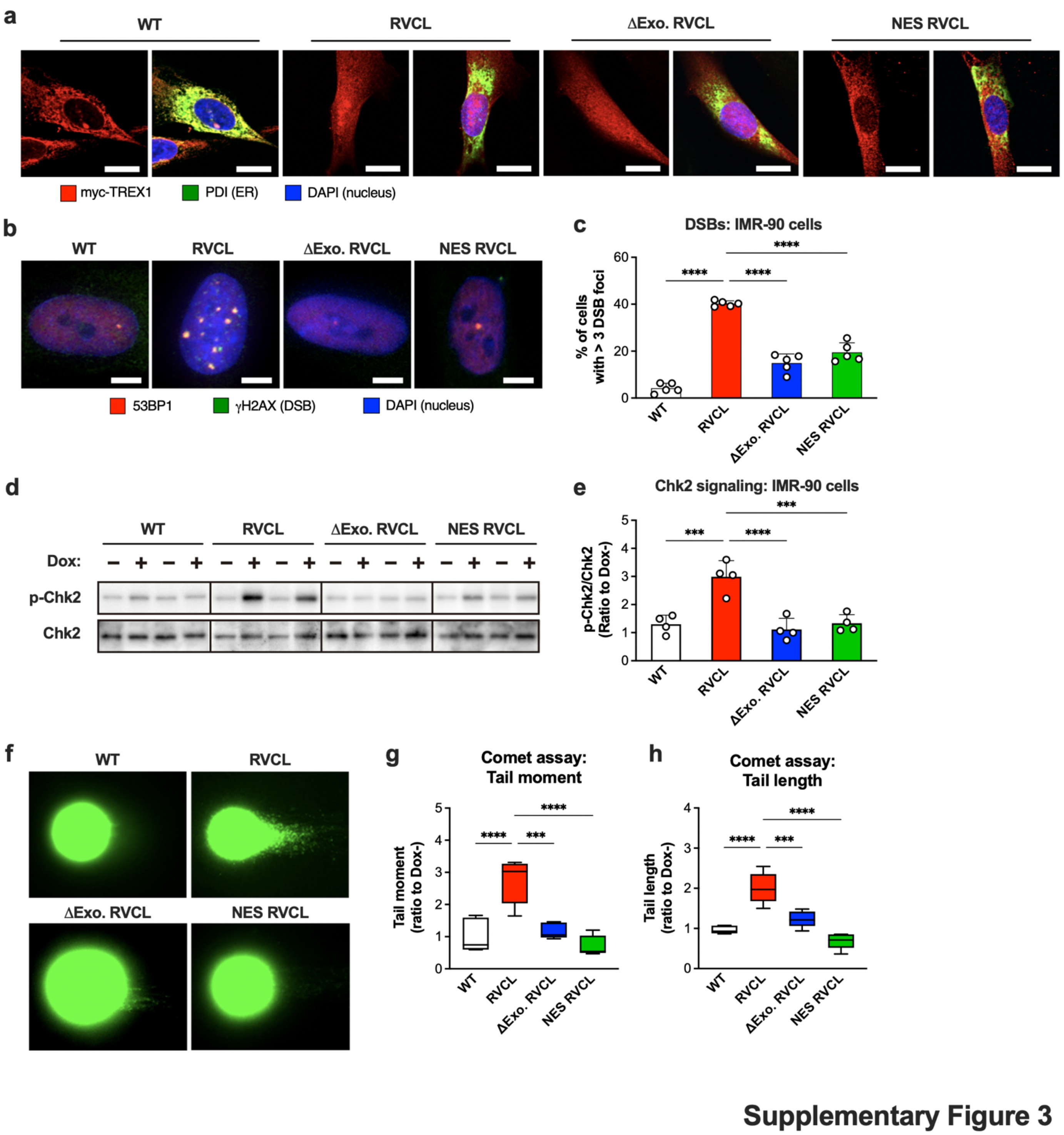

Supplementary Figure 3. RVCL mutant TREX1 causes DNA damage via its exonuclease activity and nuclear localization. (**a**) Representative fluorescence microscopy images of IMR-90 cells expressing myc-tagged TREX1 with immunostaining for myc (TREX1), PDI (ER), and DAPI (nucleus). Scale bar = 15 μm. (**b**) Representative fluorescence microscopy images of IMR-90 cells with immunostaining for 53BP1, γH2AX, and DAPI at 48 hours after addition of doxycycline. Scale bar = 10 μm. (**c**) Quantitation of percent of cells with

more than 3 53BP1/γH2AX (DSB) foci from (b). (**d**) Representative Western blot of IMR-90 cells before and after doxycycline-induced expression of WT TREX1, RVCL TREX1, or RVCL TREX1 with a nuclear export signal (NES RVCL) or lacking enzymatic activity (ΔExo RVCL), followed by immunoblotting for p-Chk2 and Chk2. Vertical lines in the image indicate that the images are from different blots. (**e**) Quantitation of the ratio of p-Chk2 to Chk2 from (d). (**f–h**) Representative image of comet assay (f) with quantitation of tail length (g) and tail moment (h). Data in (a, b, d, and f) are representative of 3–4 independent experiments. Data in (c, e, g and h) show mean ± SD, and were analyzed by ANOVA with Bonferroni post hoc comparison. Source data are provided as a Source Data file. *** $P < 0.001$; **** $P < 0.0001$.

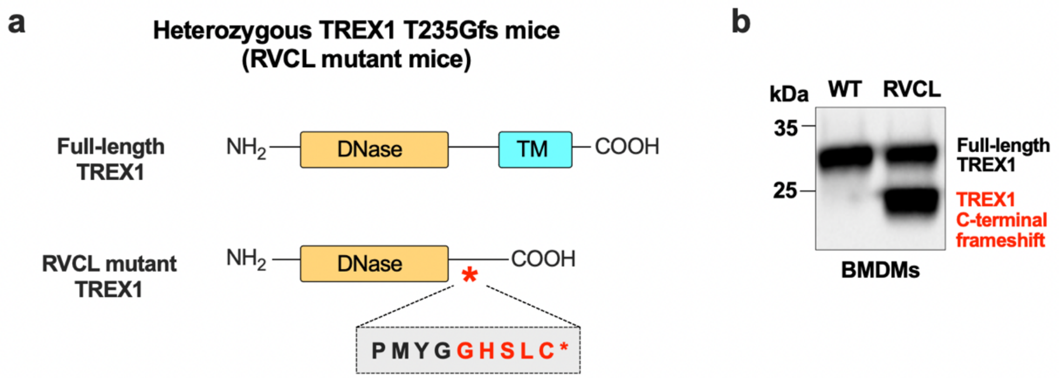

Supplementary Figure 4

Supplementary Figure 4. Heterozygous RVCL mutant mice express both full-length and C-terminally truncated TREX1. (**a**) A schematic of the amino acid sequence of the RVCL-causing TREX1 C-terminal frameshift mutation at position 235, which results in a truncated form of the protein with a missing transmembrane domain (T235Gfs in mouse TREX1, and V235Gfs in human TREX1). (**b**) A representative Western blot of mouse TREX1 protein expression from bone marrow-derived macrophages (BMDMs) of WT littermate control and heterozygous TREX1 T235Gfs mice (RVCL), that express RVCL mutant TREX1 from the endogenous locus, under control of the endogenous promoter. Data in (b) are representative of 3 independent experiments. Source data are provided as a Source Data file.

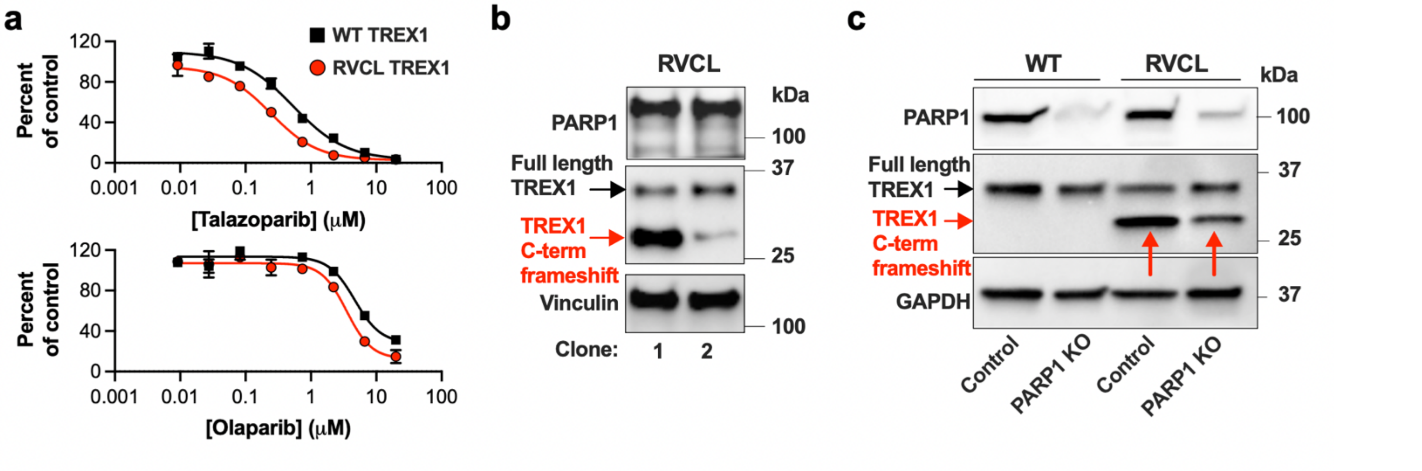

Supplementary Figure 5

Supplementary Figure 5. Heterogeneity of TREX1 expression and sensitivity of RVCL cells to PARP1 inhibition or deletion. (**a**) Dose response curves of WT and non-clonal heterozygous RVCL MEFs treated with indicated dilutions of PARP inhibitors. Data expressed as viability relative to vehicle (DMSO) treated controls. (**b**) A representative Western blot of monoclonal heterozygous RVCL MEFs immunoblotted for TREX1, PARP1, and vinculin. (**c**) A representative Western blot of WT and heterozygous RVCL MEFs transduced with a lentivirus encoding Cas9 and an sgRNA against PARP1 or a scrambled control sgRNA with immunoblotting for PARP1, TREX1, and GAPDH. Data in (a) are from a screen with 3 biologically independent samples. Data in (b-c) are representative of 3 independent experiments. Source data are provided as a Source Data file.

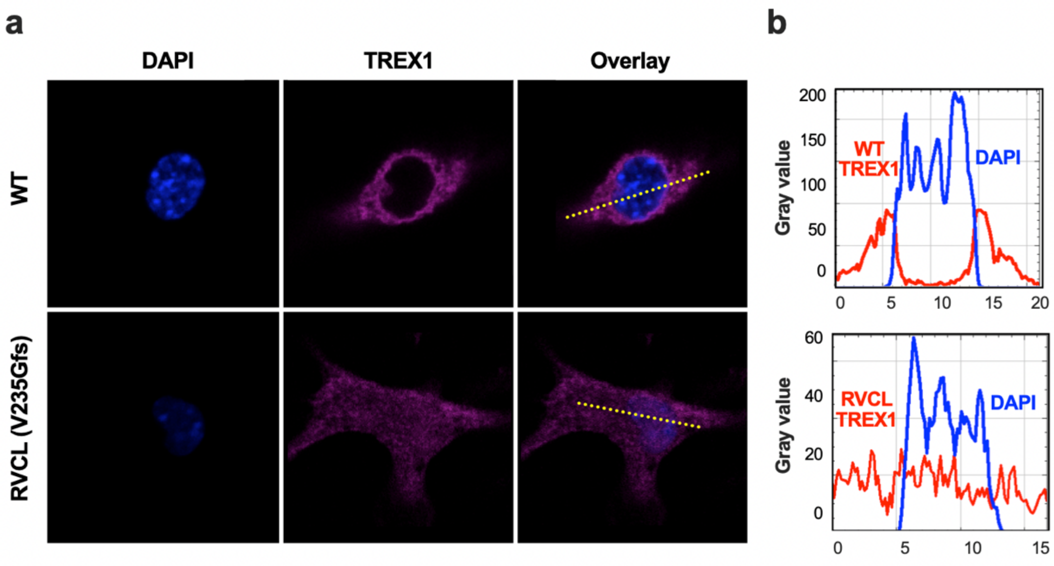

Supplementary Figure 6

Supplementary Figure 6. Aberrant nuclear localization of TREX1 in primary bone marrow-derived macrophages from RVCL mice. (**a**) Representative confocal images of TREX1 localization and DAPI in WT or TREX1 V235Gfs (RVCL) bone marrow-derived macrophages from mice expressing human TREX1 under control of the CAG promoter. (**b**) Representative fluorescent signal intensity of DAPI (nucleus) and TREX1 signal quantitation measured along the transverse plane of the cell (dotted yellow line) from (a). Data in (a-b) are representative of many hundreds of cells observed in at least 3 independent experiments. Source data are provided as a Source Data file.

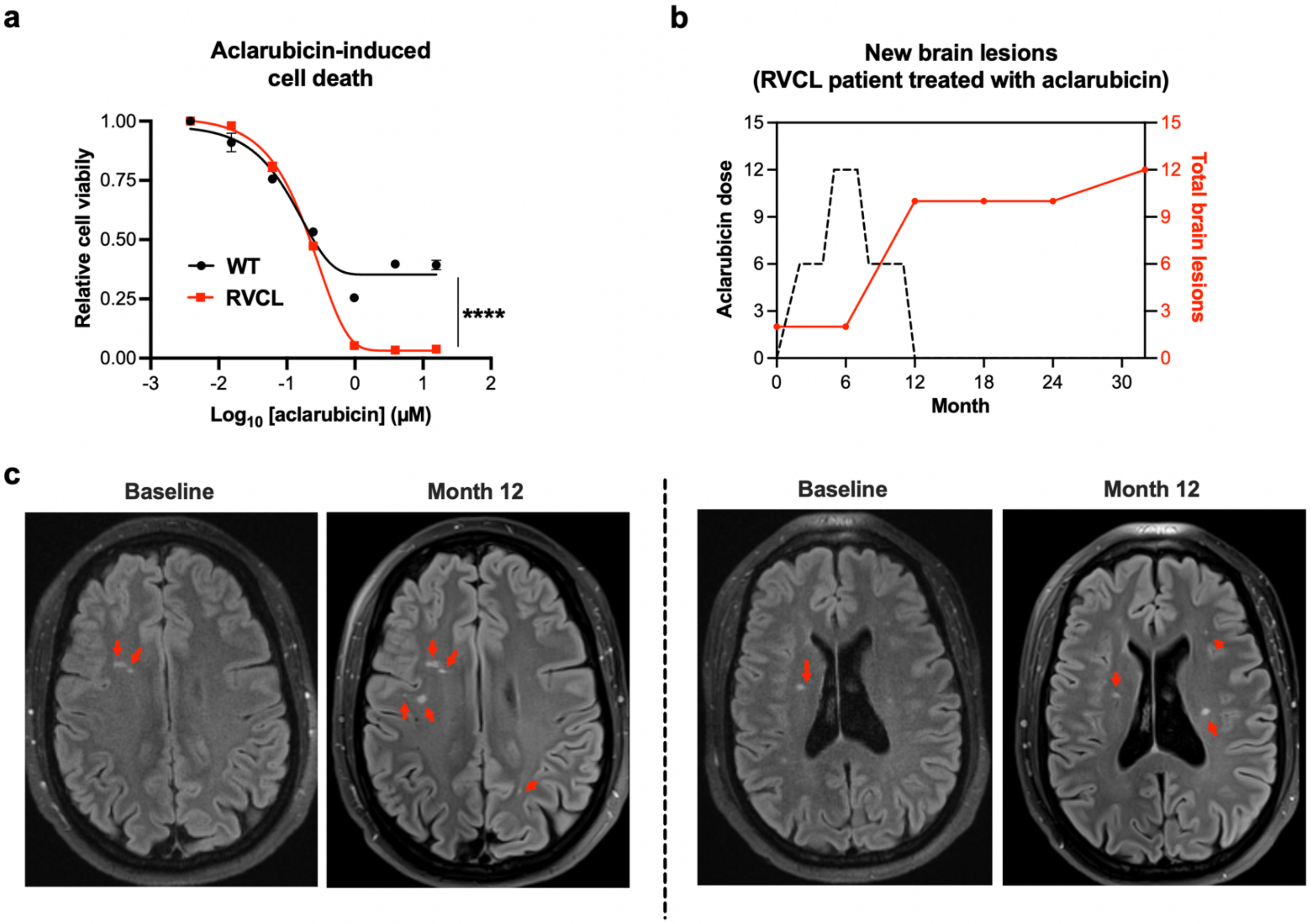

Supplementary Figure 7

Supplementary Figure 7. Sensitivity of RVCL cells and a patient with RVCL to aclarubicin. (**a**) Dose response curves of WT and RVCL MEFs treated with the indicated concentrations of aclarubicin. Data expressed as viability relative to the lowest dose of aclarubicin. (**b**) Line graph showing the total number of new brain lesions over time after a patient was treated with the indicated doses of aclarubicin. (**c**) Axial FLAIR MRI images on the patient from (b) before and after receiving aclarubicin treatment for 12 months. Brain lesions indicated with red arrow. Data in (a) are from mice expressing TREX1 out of the endogenous locus under control of the endogenous promoter. Data in (a) represent the mean ± SEM of n = 9 samples from 3 independent repeats and were analyzed by F test of the sum-of-squares. Source data are provided as a Source Data file. **** $P < 0.0001$.

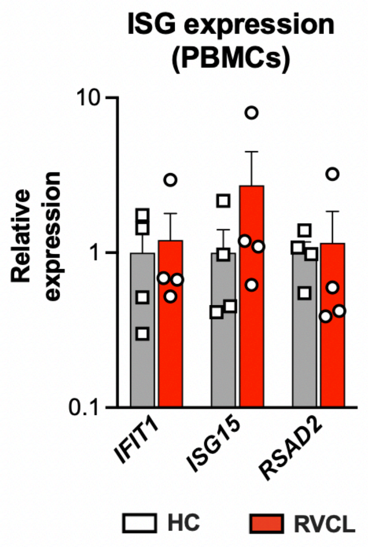

Supplementary Figure 8

Supplementary Figure 8. Interferon-stimulated gene expression in peripheral blood cells from patients with RVCL. Relative gene expression of the ISGs *IFIT1*, *ISG15* and *RSAD2* in peripheral blood mononuclear cells (PBMCs) of RVCL patients relative to those of healthy controls (HC). Data represent n = 4 independent biological replicates from experiments performed in triplicate. Source data are provided as a Source Data file.

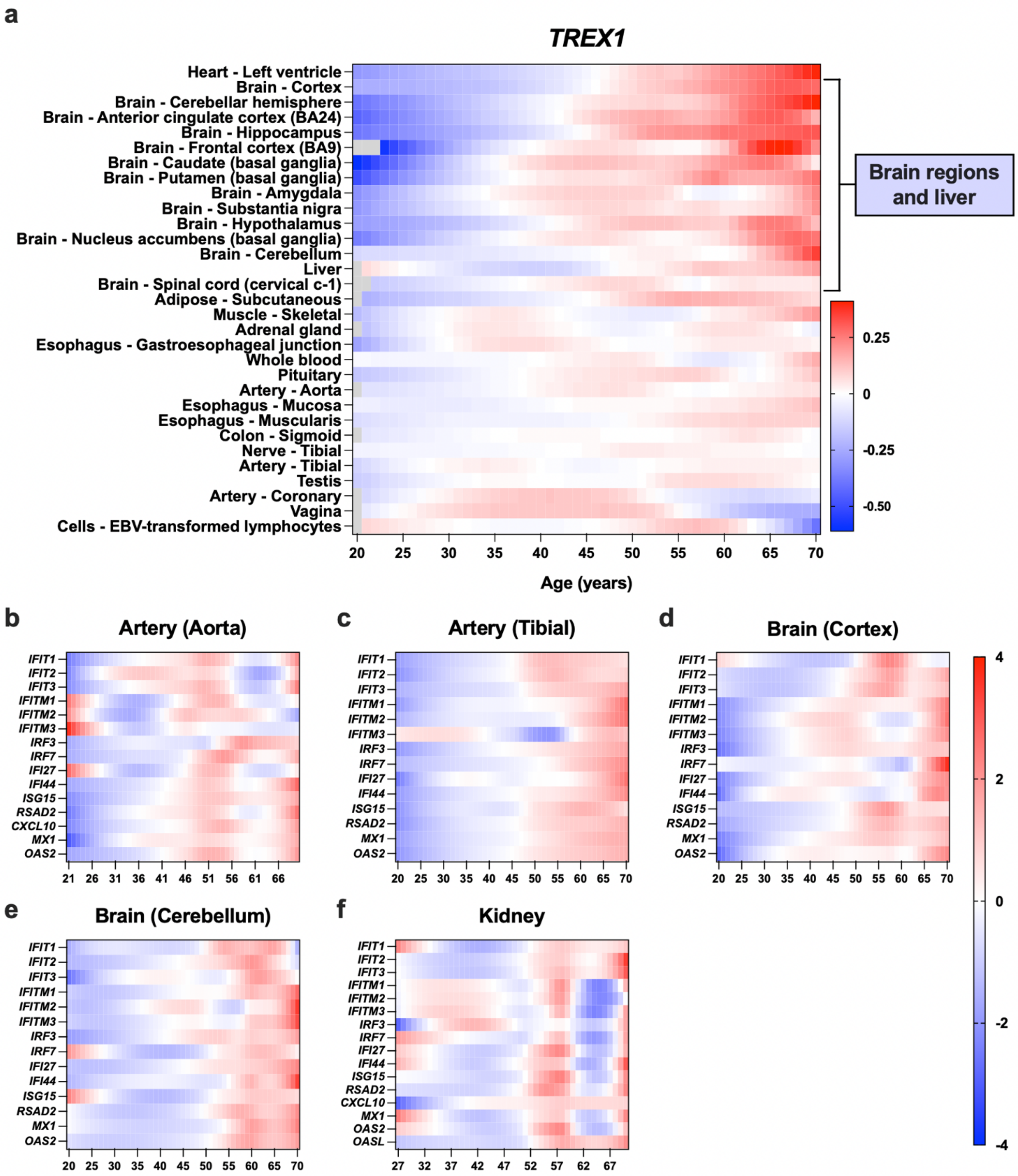

Supplementary Figure 9

Supplementary Figure 9. Changes in *TREX1* and ISG expression in human tissues with age. (**a**) Heatmap showing the relative gene expression of *TREX1* in the indicated tissues in humans at the specified ages. (**b-f**) Heatmap showing the relative gene expression of select ISGs in the indicated tissues in humans of different ages. Data are from the publicly accessible voyAGEr database with transcriptome data from the

tissues of over 700 individuals. Data are represented as the log_2 counts per million normalized to average gene expression for all ages in that tissue. Missing data is indicated in grey. Source data are provided as a Source Data file.

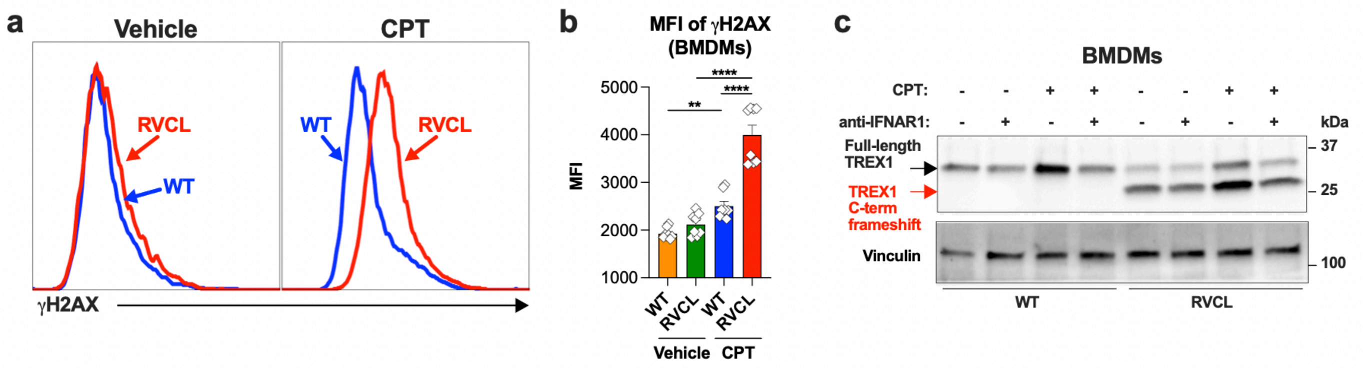

Supplementary Figure 10

Supplementary Figure 10. IFNAR1-dependent up-regulation of TREX1 in response to DNA-damaging agents. (**a**) Representative histograms of γH2AX expression of LysM-Cre-positive LSL TREX1 BMDMs treated with vehicle or CPT (1.25 μM) for 72 hours. (**b**) Quantitation of mean fluorescence intensity (MFI) of γH2AX immunostaining from (a). (**c**) Representative Western blot of BMDMs from WT or heterozygous RVCL mice after 3 days of incubation with vehicle or CPT (125 ng/mL) plus isotype or anti-IFNAR1 blocking antibody (1 μg/mL), followed by immunoblotting for TREX1 and Vinculin. RVCL mice used in (c) express RVCL mutant TREX1 from the endogenous locus, under control of the endogenous promoter. Data in (a) and (c) are representative of 3 independent experiments. Data in (b) represent the mean ± SEM of n = 9 samples from 3 independent repeats. Data in (b) were analyzed by ANOVA with Šídák's correction for multiple comparisons. Source data are provided as a Source Data file. ** $P < 0.01$; **** $P < 0.0001$.
