# Supplementary material for: Inherited C-terminal TREX1 variants disrupt homology-directed repair to cause senescence and DNA damage phenotypes in Drosophila, mice, and humans
Source: Nat Commun. 2024 Jun 1;15:4696. doi: 10.1038/s41467-024-49066-7 (PMC11144269; doi:10.1038/s41467-024-49066-7)
Supplement: Supplementary file 3 — Description of Additional Supplementary Files [file 41467_2024_49066_MOESM3_ESM.pdf]

Description of Additional Supplementary Files

File Name: Supplementary Data 1
Description: RNAi screening using RVCL TREX1-expressing Drosophila: Primary RNAi screening results and phenotypic scores in secondary screening.

File Name: Supplementary Data 2
Description: Multiplex assay of 83 proteins in the sera of RVCL patients and healthy controls.

File Name: Supplementary Data 3
Description: Primers for TREX1 plasmid mutagenesis.

File Name: Supplementary Data 4
Description: Sequences utilized in qPCR assays.
