# Supplementary material for: Inherited C-terminal TREX1 variants disrupt homology-directed repair to cause senescence and DNA damage phenotypes in Drosophila, mice, and humans
Source: Nat Commun. 2024 Jun 1;15:4696. doi: 10.1038/s41467-024-49066-7 (PMC11144269; doi:10.1038/s41467-024-49066-7)
Supplement: Supplementary file 8 — Reporting Summary [file 41467_2024_49066_MOESM8_ESM.pdf]

nature portfolio

Corresponding author(s): Jonathan J. Miner

Last updated by author(s): Mar 26, 2024

Reporting Summary

Nature Portfolio wishes to improve the reproducibility of the work that we publish. This form provides structure for consistency and transparency in reporting. For further information on Nature Portfolio policies, see our Editorial Policies and the Editorial Policy Checklist.

Statistics

For all statistical analyses, confirm that the following items are present in the figure legend, table legend, main text, or Methods section.

n/a	Confirmed	
☐	☒	The exact sample size (*n*) for each experimental group/condition, given as a discrete number and unit of measurement
☐	☒	A statement on whether measurements were taken from distinct samples or whether the same sample was measured repeatedly
☐	☒	The statistical test(s) used AND whether they are one- or two-sided<br>*Only common tests should be described solely by name; describe more complex techniques in the Methods section.*
☐	☒	A description of all covariates tested
☐	☒	A description of any assumptions or corrections, such as tests of normality and adjustment for multiple comparisons
☐	☒	A full description of the statistical parameters including central tendency (e.g. means) or other basic estimates (e.g. regression coefficient) AND variation (e.g. standard deviation) or associated estimates of uncertainty (e.g. confidence intervals)
☐	☒	For null hypothesis testing, the test statistic (e.g. F, t, r) with confidence intervals, effect sizes, degrees of freedom and P value noted<br>*Give P values as exact values whenever suitable.*
☒	☐	For Bayesian analysis, information on the choice of priors and Markov chain Monte Carlo settings
☒	☐	For hierarchical and complex designs, identification of the appropriate level for tests and full reporting of outcomes
☐	☒	Estimates of effect sizes (e.g. Cohen's *d*, Pearson's *r*), indicating how they were calculated

Our web collection on statistics for biologists contains articles on many of the points above.

Software and code

Policy information about availability of computer code

Data collection	N/A
Data analysis	Graphpad Prism, Version 10.0.03. FIJI, flynotyper

For manuscripts utilizing custom algorithms or software that are central to the research but not yet described in published literature, software must be made available to editors and reviewers. We strongly encourage code deposition in a community repository (e.g. GitHub). See the Nature Portfolio guidelines for submitting code & software for further information.

Data

Policy information about availability of data

All manuscripts must include a data availability statement. This statement should provide the following information, where applicable:

- Accession codes, unique identifiers, or web links for publicly available datasets
- A description of any restrictions on data availability
- For clinical datasets or third party data, please ensure that the statement adheres to our policy

RNA-seq data uploaded. DDBJ Sequence Read Archive.
698 (https://ddbj.nig.ac.jp/DRASearch/) (accession numbers DRA016748). All raw data will be made available on request. Mouse lines will also be shared upon request. Accession code for mass spectrometry experiments will be available before publication.

Research involving human participants, their data, or biological material

Policy information about studies with human participants or human data. See also policy information about sex, gender (identity/presentation), and sexual orientation and race, ethnicity and racism.

Reporting on sex and gender	All human serum samples were anonymous, therefore there was no sex or gender indicated. These serum samples were IRB exempt. MRI imaging result was de-identifed as part of the IRB-approved REVEAL study.
Reporting on race, ethnicity, or other socially relevant groupings	N/A
Population characteristics	N/A
Recruitment	N/A
Ethics oversight	IRB approvals and exemptions are indicated in the methods section.

Note that full information on the approval of the study protocol must also be provided in the manuscript.

Field-specific reporting

Please select the one below that is the best fit for your research. If you are not sure, read the appropriate sections before making your selection.

☒ Life sciences ☐ Behavioural & social sciences ☐ Ecological, evolutionary & environmental sciences

For a reference copy of the document with all sections, see nature.com/documents/nr-reporting-summary-flat.pdf

Life sciences study design

All studies must disclose on these points even when the disclosure is negative.

Sample size	Sample size was determined by a power calculation.
Data exclusions	No data were excluded.
Replication	All experiments were repeated in at least 2 or 3 independent experiments with multiple biological replicates as indicated in the figure legends
Randomization	Human and mouse samples were selected randomly without exclusion of any sample.
Blinding	Histology was assessed with a blinded histologist

Reporting for specific materials, systems and methods

We require information from authors about some types of materials, experimental systems and methods used in many studies. Here, indicate whether each material, system or method listed is relevant to your study. If you are not sure if a list item applies to your research, read the appropriate section before selecting a response.

Materials & experimental systems

n/a	Involved in the study	
☐	☒	Antibodies
☐	☒	Eukaryotic cell lines
☒	☐	Palaeontology and archaeology
☐	☒	Animals and other organisms
☐	☒	Clinical data
☒	☐	Dual use research of concern
☒	☐	Plants

Methods

n/a	Involved in the study	
☒	☐	ChIP-seq
☐	☒	Flow cytometry
☒	☐	MRI-based neuroimaging

Antibodies

Antibodies used	Primary antibodies used in this study: Myc-tag (MBL, 562), 53BP1 (Novus Biologicals, NB100-904), γH2AX (phospho S139) (Abcam, 26350), ATM (phospho S1981) (Rockland, 200-301-400), γH2AX (CST, 2577), H2AX (CST, 2595), GAPDH (CST, 2118), TREX1 (BD, 611986), PARP1 (CST, 9542) and H327kac (CST, 8173), Myc (MBL, 562), Tubulin (Sigma, T9026), Chk2 (CST, 2662), p-Chk2 (T68) (CST,

2197), ATM (abcam, ab32420), p-ATM (S1981) (abcam, ab81292), γH2AX (CST, 9718) HA (CST, 2367), γH2AX (abcam, ab26350),ER-associated protein disulfide isomerase (PDI) (Thermo Fisher, S34253), HA (CST, 2367S), CD45 (BV605, BioLegend, 30-F11), CD4 (BV421, BioLegend, GK1.5), CD8a (PerCP/Cy5.5, BioLegend, 53-6.7), CD19 (FITC, BioLegend, 6D5), NK1.1 (PE, BioLegend, PK136), CD11c (AF488, BioLegend, N418), CD11b (BV510, BioLegend, M1/70), MHCII (PE, BioLegend, M5/114.15.2), Ly6G (PerCP/Cy5.5, BioLegend 1A8), Ly6C(BV421, BioLegend, HK1.4), and F4/80 (AF700, BioLegend, BM8).

Secondary antibodies used in this study:
goat anti-rabbit IgG (H+L) Highly Cross-Absorbed Secondary Antibody Alexa Fluor 488 (Thermo Fisher, A-11034), 568 (Thermo Fisher, A-11036), or goat anti-mouse IgG (H+L) Highly Cross-Absorbed Secondary Antibody Alexa Fluor 488 (Thermo Fisher, A-11029), Alexa Fluor 568 (Thermo Fisher, A-11031), Horseradish peroxidase conjugated secondary anti-rabbit antibody (CST, 7076S), Horseradish peroxidase conjugated secondary anti-mouse antibody (CST, 7074S), AF488 donkey anti-rabbit IgG (Invitrogen, A-21206) and AF647 donkey anti-mouse IgG (Invitrogen, A-31571).

Validation	Antibodies were validated either in knockout cells or by Western blot indicating proteins of appropriate molecular weights.

Eukaryotic cell lines

Policy information about cell lines and Sex and Gender in Research

Cell line source(s)	293T (ATCC, CRL-3216), IMR-90 (ATCC, CCL-186),
Authentication	Cells came directly for ATCC and were authenticated at ATCC
Mycoplasma contamination	All cell lines tested negative for mycoplasma
Commonly misidentified lines (See ICLAC register)	N/A

Animals and other research organisms

Policy information about studies involving animals; ARRIVE guidelines recommended for reporting animal research, and Sex and Gender in Research

Laboratory animals	Mus musculus (C57BL/6J),
Wild animals	N/A
Reporting on sex	Equal numbers of both sexes were used in experiments.
Field-collected samples	N/A
Ethics oversight	All protocols for animal studies were approved by the Institutional Animal Care and Use Committees (IACUC) or Institutional Review Boards (IRB) or determined to be IRB-exempt at the respective institutions.

Note that full information on the approval of the study protocol must also be provided in the manuscript.

Clinical data

Policy information about clinical studies
All manuscripts should comply with the ICMJE guidelines for publication of clinical research and a completed CONSORT checklist must be included with all submissions.

Clinical trial registration	N/A
Study protocol	IRB-exemptions and approved protocols indicated in manuscript, otherwise all human samples were anonymous and exempt.
Data collection	N/A
Outcomes	N/A

Plants

Seed stocks

N/A

Novel plant genotypes

N/A

Authentication

N/A

Flow Cytometry

Plots

Confirm that:

☒ The axis labels state the marker and fluorochrome used (e.g. CD4-FITC).

☒ The axis scales are clearly visible. Include numbers along axes only for bottom left plot of group (a 'group' is an analysis of identical markers).

☒ All plots are contour plots with outliers or pseudocolor plots.

☒ A numerical value for number of cells or percentage (with statistics) is provided.

Methodology

Sample preparation

5x10^6 marrow cells were cultured for 37°C in 5% CO_2 in 10-cm Petri dishes with D10 with 40ng/ml of macrophage colony-stimulating factor (M-CSF) (PeproTech, 315-02). On day 3 macrophages were fed with 5mL complete DMEM containing 40 ng/mL M-CSF.

Murine embryonic fibroblasts were isolated from embryos and immortalized via sequential passage in complete DMEM.

To assess cellular viability, cell suspensions were washed with DPBS to remove residual FBS then stained with Zombie NIR™ (BioLegend, 423106) in DPBS for 15 minutes on ice. Cells were washed and fixed in 4% PFA for 10 minutes at room temperature. After fixation, cells were permeabilized in 90% methanol for 15 minutes on ice. Cells were washed to remove residual methanol, and stained for gammaH2AX (CST, 9718) and HA (CST, 2367) in FACS buffer for 1 hr on ice. Fc-mediated interactions were blocked by incubating cell suspensions with purified rat anti-mouse CD16/32 (BD Biosciences, 553142) in FACS buffer during primary staining. Fixed cells were then stained with the fluorescently labeled secondary antibodies AF488 donkey anti-rabbit IgG (Invitrogen, A-21206) and AF647 donkey anti-mouse IgG (Invitrogen, A-31571).

For flow cytometry studies of splenocytes, spleens were mashed through a 70 µm strainer and washed in PBS. Red blood cells were lysed in ACK Lysing Buffer (Gibco, A10492-01) before staining with Zombie NIR in PBS for 15 minutes on ice. Cells were then stained for CD45 (BV605, BioLegend, 30-F11), CD4 (BV421, BioLegend, GK1.5), CD8a (PerCP/Cy5.5, BioLegend, 53-6.7), CD19 (FITC, BioLegend, 6D5), and NK1.1 (PE, BioLegend, PK136), or CD45 (BV605, BioLegend, 30-F11), CD11c (AF488, BioLegend, N418), CD11b (BV510, BioLegend, M1/70), MHCII (PE, BioLegend, M5/114.15.2), Ly6G (PerCP/Cy5.5, BioLegend 1A8), Ly6C(BV421, BioLegend, HK1.4), and F4/80 (AF700, BioLegend, BM8).

Instrument

Attune NxT Flow Cytometer (Thermo Fisher) , LSR II (BD Biosciences)

Software

FlowJo™ v10 software (FlowJo LLC)

Cell population abundance

N/A (single cell populations)

Gating strategy

Single populations were gated / single color flow

☒ Tick this box to confirm that a figure exemplifying the gating strategy is provided in the Supplementary Information.
